# SMART-ly Managing Type 1 Diabetes - Modifying Glucose Metabolism With an Online Mind-Body Intervention: A Feasibility and Pilot Study

**DOI:** 10.3389/fcdhc.2022.802461

**Published:** 2022-03-03

**Authors:** James E. Stahl, Hima R. Ammana, Leigh Kwak, Richard J. Comi

**Affiliations:** ^1^ Section General Internal Medicine, Dartmouth-Hitchcock Medical Center, Lebanon, NH, United States; ^2^ Department of Medicine, The Geisel School of Medicine at Dartmouth, Hanover, NH, United States; ^3^ The Dartmouth Institute for Health Policy & Clinical Practice, Lebanon, NH, United States; ^4^ Section Endocrinology, Dartmouth-Hitchcock Medical Center, Lebanon, NH, United States

**Keywords:** mind-body medicine, type 1 diabetes, stress management, resiliency, mindfulness

## Abstract

**Objective:**

Managing type 1 diabetes is stressful. Stress physiology influences glucose metabolism. Continuous glucose monitors allow us to track glucose variability in the real-world environment. Managing stress and cultivating resiliency should improve diabetes management and reduce glucose variability.

**Research Design and Methods:**

The study was designed as a randomized prospective cohort pre-post study with wait time control. Participants were adult type 1 diabetes patients who used a continuous glucose monitor and recruited from an academic endocrinology practice. The intervention was the Stress Management and Resiliency Training (SMART) program conducted over 8 sessions over web-based video conference software. The main outcome measures were Glucose variability, the Diabetes Self-Management questionnaire (DSMQ),Short-Form Six-Dimension (SF-6D), and the Connor-Davidson Resiliency (CD-RSIC) instrument.

**Results:**

There was statistically significant improvement in participants DSMQ and CD RISC scores though the SF-6D did not change. Participants under age 50 years-old showed a statistically significant reduction in average glucose (p = .03) and Glucose Management Index (GMI) (p = .02). Participants also had reduced percentage of time high and increased time in range though this did not reach statistical significance. The participants found doing the intervention online acceptable if not always ideal.

**Conclusions:**

An 8-session stress management and resiliency training program reduced diabetes related stress and improved resiliency and reduced average blood glucose and GMI in those under 50 years-old.

**Clinical Trial Registration:**

ClinicalTrials.gov, identifier NCT04944264.

## Highlights

- Without additional medication management, this Mind-Body intervention may improve glucose control in patients with type 1 diabetes
- This Mind-Body intervention improves quality of life in patients with type 1 diabetes
- This intervention can be delivered on-line

## Introduction

Managing type 1 diabetes is stressful. Diabetes is a chronic illness that relies on self-care. Decisions about diet, exercise, and dose of medication must be made multiple times a day. In Type 1 diabetes, the patient provides all or nearly all the insulin required to control their metabolism, placing greater emphasis on correct medication decisions than in type 2 diabetes. Even patients with sensor directed insulin pumps must make the decisions about meal dosing several times a day. Patients with Type 1 diabetes must balance these self-care requirements with the usual stressors of daily life. Several studies have documented decreased quality of life for people with Type 1 diabetes when compared to those without diabetes. Diabetes specific measures of quality of life show direct associations of worsening life quality with worsening control or presence of diabetes-related complications (1–3).

Stress is a mind-body phenomenon. Stress creates a cascade of effects touching on every system in the body, including the cardiovascular, neurologic, and metabolic systems. The stress response activates the hypothalamic-pituitary-adrenal (HPA) axis resulting in the release of cortico-releasing hormone (CRH) and subsequently adrenocorticotropic hormone (ACTH) from the pituitary which in turn drives the release of stress hormones, such as glucocorticoids. Glucocorticoids stimulate (glycogenolysis) in the liver, sympathetic nervous system mediated vasoconstriction, proteolysis and lipolysis and suppress innate immunity, reproductive function, and bone and muscle growth as well as changes in mood, e.g., depression. This response is useful in the short term but pathogenic if prolonged. For patients with both type 1 and type 2 diabetes, the stress surrounding the management of their disease, diabetic distress (4, 5), can create a viscous cycle when trying to manage their blood sugar. Prior studies have indicate that training to reduce stress can have a positive impact on both quality of life and the degree of metabolic control of patients with diabetes (6).

New tools are changing the landscape of diabetes care. Continuous glucose monitors provide real time feedback to the patient, informing their medication, diet and exercise decisions (7). They also provide new parameters to assess diabetes control – the Glucose Management Indicator (GMI) which provides an estimate of average control similar to the familiar hemoglobin A1c (HbA1c), Time in Range (TIR) which provides the percentage of time spent within certain glucose concentrations (usually 100-180) and estimates of variation – coefficient of variation (CV) or standard deviation (SD),both contribute to cardiovascular risk. Although only the HbA1c has been directly tied to risk of microvascular complications, it is likely that these other parameters particularly those related to variability in control are related of microvascular risk (8, 9).

The Stress Management and Resiliency Training (SMART) training program (developed by the Benson-Henry Institute for Mind Body Medicine at Massachusetts General Hospital) is a comprehensive well-validated successful stress management program designed to reduce stress and increase resiliency in response to stress (10).

However, it has not been specifically examined in Type 1 diabetes. The use of new sensor technology makes it possible to look in greater detail at the impact of stress management on diabetes glucose control. Finally, due to the restrictions of the novel coronavirus SARS-CoV2 (COVID-19) pandemic, we delivered the SMART program *via* an internet platform which allows much greater potential access for patients. Therefore, we devised a study to look at the impact of the SMART stress management program in patients with Type 1 diabetes for impact on quality of life, glucose control parameters recorded by sensors and delivered on an online video conference platform. We hypothesized that the online course would deliver similar impacts on quality of life as has been seen in the past from on-site courses and applications of the SMART program. We also hypothesize that the intervention would reduce glucose variability as well as average glucose and time in range.

## Research Design and Methods

### Participant Recruitment

Recruitment occurred through the Dartmouth-Hitchcock Medical Center (DHMC) endocrinology clinic and the endocrinologists working there. Candidates were included if they had type 1 diabetes and used a continuous glucose monitor. Candidates were excluded if they were < 21 years old and could not give informed consent. To allow for controlled analysis, on presentation at each site, participants were randomly assigned to one of two cohorts: 1) immediate start (A) and 2) delayed start (B). The immediate arm began at the next available class. The delayed start group began 4 weeks later. During their wait, this group was offered usual care.

### Description of Intervention

The Stress Management and Resiliency Training (SMART) program (bensonhenryinstitute.org) is well validated comprehensive stress management program. It is designed to cultivate both the early recognition of stress in the mind and body, develop skills to mitigate stress and evoke the relaxation response and cultivate resiliency. It is an 8-session program, typically run in a live group setting, taking advantage of the opportunity to cultivate social support. It can also be run for individuals.

Mind-Body Medicine takes as a core principle that the mind and body are a unity. Psychosocial stress creates cellular stress and in turn mitochondrial oxidative stress (11). Stress causes a cascade of phenomena that result in among other things gluconeogenesis (12, 13), hence part of the reasoning for this class of intervention. The program specifically incorporates elements of training in the relaxation response, mindfulness, cognitive behavioral training, social support and prosocial behavior, positive psychology, belief and conscious expectation, exercise, diet, and sleep. The SMART program uses a top-down approach (14), training the prefrontal cortex to downregulate among other things the stress response in the amygdala which in turn creates a positive cascade of events mediated through the hormonal, cardiovascular and nervous systems to encourage healing and optimal function (15–17).

In this study’s case it was delivered *via* a videoconferencing platform. This was done both as a means of testing delivering this service in a rural setting where patient might be geographically distant or isolated and to accommodate the need for social distancing during the COVID-19 pandemic.

### Study Design

This pilot was designed as a prospective cohort pre-post intervention study with participants randomized to an immediate start or wait time control. The study was approved by the Committee for the Protection of Human Subjects at Dartmouth Hitchcock Medical Center and Dartmouth College. All participants provided written informed consent.

### Hypothesis

We hypothesized that the course on videoconferencing platform would deliver similar effects on quality of life as has been seen in the past from on-site courses, and that the intervention would reduce glucose variability and improve resiliency.

### Outcome Measures

Demographic data was collected during enrollment. The outcome measures collected were the mean glucose, glucose standard deviation (SD), the Glucose Management Index (GMI), systolic blood pressure (sBP), diastolic blood pressure (dBP), glycated hemoglobin (HbA1c), the Short-Form Six-Dimension (SF-6D), the Diabetes Self-Management Questionnaire (DSMQ), the Connor-Davidson Resilience Scale (CD-RISC).

#### Continuous Glucose Monitor Related Outcomes

The GMI (Glucose Management Index) approximates the laboratory HbA1C level expected based on average glucose measured using continuous glucose monitoring (CGM) values. Average glucose is derived from at least 12 days of CGM data. The GMI may be similar to, higher than or lower than the laboratory HbA1C. The glucose standard deviation is a measure of the variability of the glucose measured by the CGM.

#### SF-6D

The SF-6D is a preference-based measure of health with that uses six-dimensions to classify health status: physical functioning, role functioning, social functioning, pain and discomfort, mental health and vitality (18, 19). It is derived from the SF-36 Health Survey, a widely used generic health profile developed in the US. Participants select one of the levels (ranging from 4 to 6 levels depending on the dimension) which best describes their current health status. The scoring algorithm of preference-based values in different levels (SF-6D) was mapped to single composite score. This algorithm was derived from the work at the University of Sheffield. The authors have registered in the University of Sheffield website.

#### The DSMQ

The Diabetes Self-Management Questionnaire (DSMQ) is a well validated measure to assess diabetes self-care activities (20). Diabetes self-care activities in turn are highly correlated to diabetic distress and glycemic control (21–23). The scale has 4 main domains: medication adherence, glucose monitoring, physical activity and healthcare system contact related to glycemic control, e.g., a clinical interaction related to medication management.

The DSMQ consists of 16 items formulated as behavioral descriptions from the person’s point of view. For example, respondents rate the extent to which each description applies to them on a four-point Likert scale (3 –’applies to me very much’ to 0 –’does not apply to me’), referring to the previous eight weeks. Higher scores indicate more desirable self-management behavior. The 9 negatively framed items require reverse scoring.

#### The CD-RISC

CD-RISC (24) comprises 25 statements covering 17 domains relevant to stress and resiliency as experienced by the participant over the past month. These include adaptability, self-efficacy, sense of control, purpose, focus, social support, humor, agency, optimism, and others. The response scale has a 5-point range: 0 (not true at all), 1 (rarely true), 2 (sometimes true), 3 (often true), and 4 (true nearly all the time). Scores are added up to a maximum score of 100. The higher the score the higher resilience.

#### Qualitative questions

At study completion, participants were surveyed with open-ended questions asking what they found to be barriers and facilitators in participating in the study and what did they value or not value about participating in the study.

### Timing of Evaluations

Participants completed the evaluations at three points throughout the course of the study: at T=0 (study start); T=1 (one month after starting classes); T=3 (one month following completion of 8 weeks of classes). The wait-time participants began their evaluations and instruction one month after the immediate-start group.

### Statistical Analyses

Descriptive analysis of continuous variables included median and interquartile range (IQR), or mean and standard deviation (SD) as appropriate. Categorical variables were reported as counts and percentages. Baseline characteristics were compared between the two groups using Chi-square test or Fisher’s exact test where appropriate for categorical variables and t-test or ANOVA for continuous variables for all enrolled participants. Linear regression analysis was used analyze and explore the effect of independent variables on the outcome measures.

Computations were performed using (JMP15, SAS Institute Inc., Cary, NC). Statistical significance was defined as p < 0.05 based on a two-sided hypothesis test with no adjustments made for multiple comparisons.

Sample size is an *a priori* best guess estimate of the number of participants needed to detect a hypothesized difference. As this was a pilot study, testing for both effect and feasibility in a complex changing environment this was not a relevant requirement.

## Results

**Figure 1:**
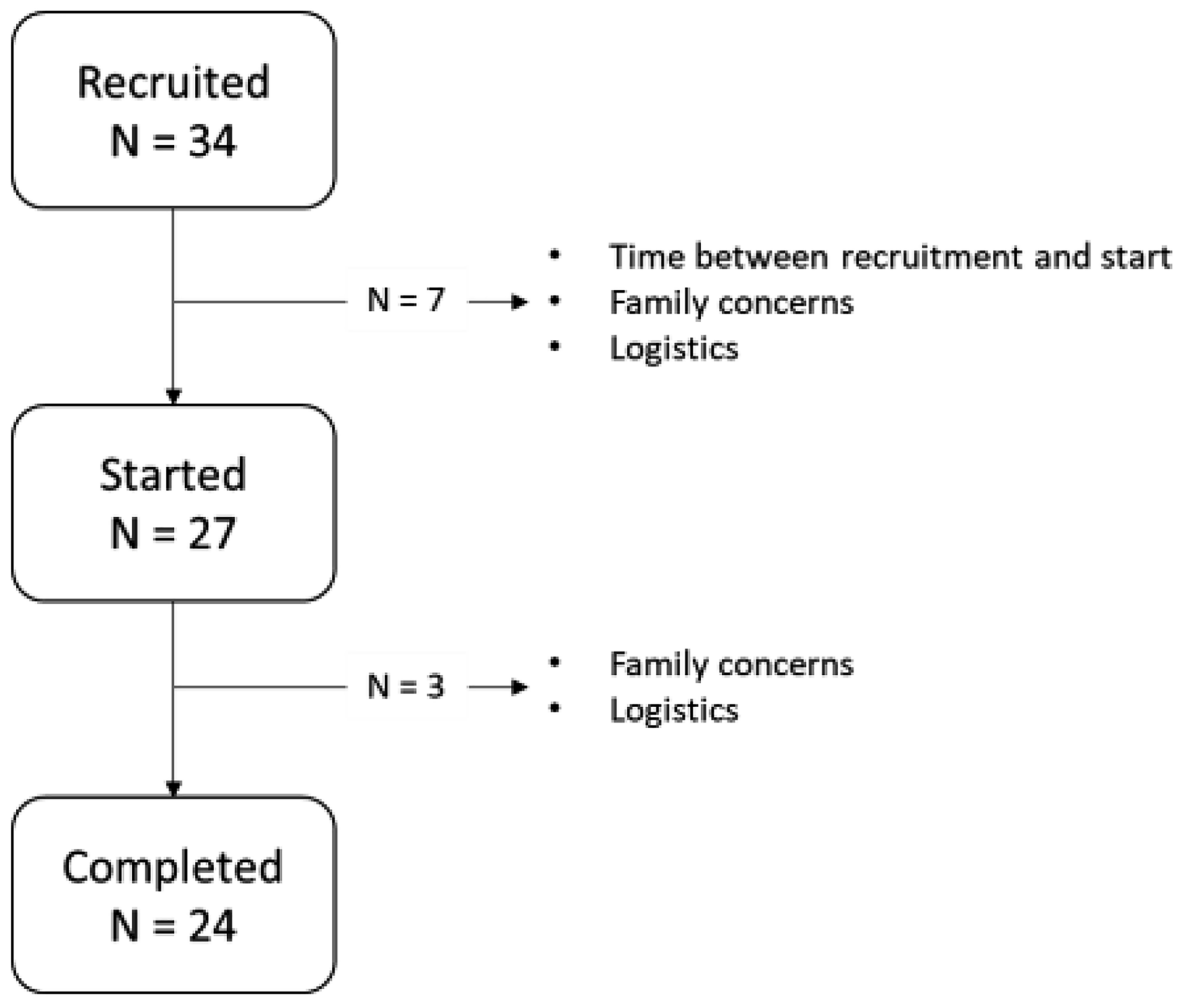
Recruitment.

A total of 34 participants were contacted and enrolled. Five dropped out before the study started. The stated reasons being extent of time between recruitment and study start and personal scheduling and logistical concerns. Twenty-seven began the study, 3 dropped out because of due to family and logistical reasons (see **Figure 1**). The median age for the group on enrollment was 61 and included more women (77%) than men (23%). The immediate start and wait time control groups were statistically indistinguishable from each other (**Table 1**).

**Table 1:** Demographics.

		All	Immediate start	Wait control	p
**n**			14 (52%)	13 (48%)	
**Age (years)**	Mean (SD)	58.85 (16)	58.8 (16.6)	59.4 (14.8)	0.9
	Median	64	64	65	
**Gender**	(% Female)	21 (77%)	10 (70%)	12 (90%)	0.15
**Race**	American Indian or Alaska Native (%)	1 (4%)	1 (7%)	0 (0%)	
	Asian (%)	1 (4%)	1 (7%)	0 (0%)	
	Black or African-American, (%)	0 (0%)	0 (0%)	0 (0%)	
	Native Hawaiian or Other Pacific Islander (%)	0 (0%)	0 (0%)	0 (0%)	
	White (%)	25 (92%)	12 (86%)	13 (100%)	
	Other (%)	2 (8%)	2 (14%)	0 (0%)	
				P for ethnicity	.35
**Median Income (SD)**	($K)*	58 (20)	57 (21)	58 (21)	0.35
**Median HS Grad (SD)**	(% High School graduation)*	94 (3.6)	93.1 (4.5)	94.4 (2)	0.75

*based on zip code census data, SD indicates standard deviation.

Because our main physiologic outcome measures were based on CGM data we needed to determine their measurement variability. This was in case we had to control for this in our analysis. Our patients used 3 different types of CGM which did indeed have significant measurement variability. However, this difference between types of CGM remained consistent within CGM class, across all sample times and across the CGM provided metrics. Almost a third (27%) of study patients were on insulin pumps and had been for at least 1 year, though that was not an inclusion or exclusion criterium.

After controlling for CGM, glucose variability as measured by standard deviation statistically significantly reduced (P <.05), even in this relatively small sample. The other measures had parameters in the desirable direction but did not achieve statistical significance during the period the participants were being measure in the study. Systolic and diastolic blood pressures declined for the under 50 group but did not reach statistical significance and remained stable for the older group. HgbA1c, in the context of the CGM, unfortunately was not consistently collected for the groups and was too sparse for analysis.

On subset analysis (**Table 2**) the main items found were that patients who were 50 years-old and younger (n = 5) had drops in mean blood glucose of 10 pts 161 to 151 p = .03 and GMI dropping from 7.2 to 6.9 p = .02 the rest of the data showed decreased percent times high and increased time in range (TIR), but these later did not achieve statistical significance in this small sample.

**Table 2:** Blood sugar, GMI, Percent High and Percent Time in Range in those < 50 and > 50 years old.

	Average BG		GMI		Percent high		Time in range	
Time	Age		Age		Age		Age	
	<50	>50	<50	>50	<50	>50	<50	>50
	5	19	5	19	5	19	5	19
T = 0	160.8	153.6	7.16%	6.98%	30.9%	28.5%	64.0%	67.0%
1 mo.	156.1	154.2	7.04%	6.97%	28.3%	28.2%	68.5%	65.0%
2 mo.	151.4	154.8	6.92%	6.95%	25.8%	27.8%	73.0%	70.0%
P	0.03	0.9	0.02	0.85	.2	.9	.8	.8

Qualitatively these changes were accompanied by significant improvements in DSMQ and CD-RISC. The SF6D scores remained statistically unchanged. (See **Table 3**) On subset analysis, the improvement in psychologic resiliency and the reduction in stress was driven most by improvements in the domains of humor, purpose and sense of control, and clarity of focus.

**Table 3:** Quality of life outcomes.

Measure	Sample time	n	Mean	Median	Min	Max	IQR	p difference
**DSMQ**	T0	27	34.6	34	26	48	13	
	T3	17	40.1	37	32	46	7.75	0.006
								
**SF-6D**	T0	20		0.95	0.92	1	0.02	
	T3	17		0.95	0.93	0.99	0.03	ns
								
**CD-RISC**	T0	27	50.3	59	0	98	76	
	T3	17	68.7	68	59.5	95	35.5	0.036

T0 = study start, T3 = 1 month post completion of 8-week intervention. Ns, Not significant.

### Qualitative Feedback

We asked 4 qualitative open-ended questions regarding participants’ experience with the intervention and one general comment opportunity.

Did you experience anything that made participating in the project easier or help you participate in the study? The answers broke down into 3 broad categories in order of frequency:

1) having the program online was helpful when travel or logistics were difficult

2) the handouts were helpful

3) the teacher’s compassion and humor

Did you experience any barriers or difficulties in participating with in the study? These answers broke down into the following in order of frequency:

1) Personal logistics, scheduling, and family time

2) Pre-class handouts were sometimes delayed

3) Homework sometimes felt burdensome

4) Group conversation online were sometimes difficult

5) Internet connection

6) Task assigned were sometimes not specific enough

What did you value about participating in the study? These answers broke down into the following in order of frequency:

1) Lessons learned about stress management were very valuable

2) Camaraderie with others dealing with same issues

3) Developing new tools and skills

What did you not value about participating in the study? These answers broke down into the following in order of frequency:

1) Being online versus being in person

2) Homework sometimes burdensome

3) Multiple emails from research team

4) Online Handouts

5) Personal logistics, scheduling, and family time

6) Insufficient disease specific counseling

General comments, broke down into the following in order of frequency:

1) Very helpful and gratitude for participating

2) Teachers’ skill, compassion, and humor

3) Camaraderie with peers

4) Stress over keeping up with homework

5) Preference for in person classes

## Discussion and Conclusions

The primary findings from this pilot study were two-fold. Though this study was limited in sample size and duration, the first finding was that participation in the 8 session SMART program (15) achieved measurable improvement in relevant clinical parameters for type 1 diabetes patients, specifically reducing their glucose variability in the group as a whole and reducing both average glucose and GMI in those under 50 years-old. This was true in even well controlled type 1 patients with both CGM, and insulin pumps examined in this pilot. This might indicate that younger patients may be more physiologically or psychologically flexible and responsive than their older peers. This difference may also be related to the duration the participants have lived with diabetes. The intervention also seemed to significantly reduce the stress surrounding managing diabetes and in improving their resiliency. Second, it was demonstrated that this could be achieved using an on-line version of the program. Third, improvements in psychologic resiliency stress reduction seemed to most attributable to improvements in the domains of humor, purpose and sense of control, and clarity of focus.

From a physiologic perspective, our experience shows that this intervention could be added to the armamentarium for treating diabetes, potentially make a large difference. This makes a great deal of sense in that diabetes physiology is directly influenced by stress physiology, and how it changes energy metabolism at the tissue, cellular and intra-cellular levels (12, 13, 16, 25). This seemed particularly true for the younger participants. By extension, this set of tools should also influence and improve the care of other diseases with direct stress-related metabolic changes and neuro-endo-cardiovascular feedback derangements (26, 27) such as heart disease, e.g., hypertension (28, 29), congestive heart failure (30) and pulmonary disease, e.g., asthma, chronic obstructive pulmonary disease (31).

Demonstrating the feasibility of using an online platform opens the door to much greater accessibility to these tools. Though this version of the SMART intervention was developed in response to the constraints caused by the COVID-19 pandemic, it proves the principle that the core lessons of the program are extensible to other platforms. It was interesting to see that there was an intimacy and bonding that occurred during the intervention that we had only expected with in-person groups before. The pandemic may have accelerated this process and the acceptability of these tools. However, we think it more likely that this may have been due to the structured journey the patients took during the course, where they shared personal feelings and insights in a guided fashion, perhaps much more than they would have in an *ad hoc* less focused on-line gathering. That said there were definite strengths and weakness to the online platform. On the one hand, it allowed a more geographically diverse group to gather and be formed than might have been possible otherwise. On the other hand, the participants did notice limitations in the kind and strength of their interactions that they didn’t necessarily find satisfactory. This may in part the different nature of the conversational floor and etiquette required in online interactions that is less natural for those who are internet immigrants versus internet natives (32, 33).

Overall, the project has demonstrated that it is indeed feasible to measurably modify diabetes physiology through a mind-body intervention and to do so in a way that may improve access to those with limited geographic access such as those in rural communities.

### Challenges

There were several challenges that were faced in this study. First, was that the program was run during the COVID19 pandemic and during a period of great political tumult. Both of these stress inducing external factors could have limited the amount of overall stress reduction the patients experienced. Second, another potential confounder was participants’ internet connectivity and facility with technology which was expressed in some of the qualitative feedback. Finally, one should always take care when analyzing a project depending on skilled operators. Though the core program has been manualized and study, the skill of the teacher could confound the programs generalizability.

### Implications for Future Research

The findings from the study suggest the need for larger scale randomized clinical trials powered to explore the effects of this intervention on a larger scale and further explore the barriers and facilitators of a stress management program delivered in online for which should be highly accessible to a broad population

### Strengths and Limitations

A strength of the study was the ability to take advantage of CGM and the internet.

A limitation of the study was the dropout rate which might bias the results. Ambient stress was also quite high at the time of the study – COVID-19 pandemic, presidential election, shifting most work and social activities online

## Data Availability Statement

The raw data supporting the conclusions of this article will be made available by the authors, without undue reservation.

## Ethics Statement

The studies involving human participants were reviewed and approved by Committee for the Protection of Human Subjects - Dartmouth-Hitchcock Medical Center. The patients/participants provided their written informed consent to participate in this study.

## Author Contributions

JS was responsible for study inception and design, recruitment of study sites, intervention, data interpretation and manuscript preparation. HA was responsible for recruitment of study sites and manuscript preparation. LK was responsible for recruitment of study sites and manuscript preparation. RC was responsible for study inception and design, recruitment of study sites and manuscript preparation. All authors contributed to the article and approved the submitted version.

## Funding

The project was supported with funding from the Hitchcock Foundation at Dartmouth Hitchcock Medical Center.

## Conflict of Interest

The authors declare that the research was conducted in the absence of any commercial or financial relationships that could be construed as a potential conflict of interest.

## Publisher’s Note

All claims expressed in this article are solely those of the authors and do not necessarily represent those of their affiliated organizations, or those of the publisher, the editors and the reviewers. Any product that may be evaluated in this article, or claim that may be made by its manufacturer, is not guaranteed or endorsed by the publisher.
